# The Importance of Extended Analysis Using Current Molecular Genetic Methods Based on the Example of a Cohort of 228 Patients with Hereditary Breast and Ovarian Cancer Syndrome

**DOI:** 10.3390/genes12101483

**Published:** 2021-09-24

**Authors:** Luise D. Resch, Alrun Hotz, Andreas D. Zimmer, Katalin Komlosi, Nina Singh, Andreas Tzschach, Marisa Windfuhr-Blum, Ingolf Juhasz-Boess, Thalia Erbes, Judith Fischer, Svenja Alter

**Affiliations:** 1 Medical Center, Faculty of Medicine, Institute of Human Genetics, University of Freiburg, 79106 Freiburg, Germany; luiseresch@gmail.com (L.D.R.); alrun.hotz@uniklinik-freiburg.de (A.H.); andreas.zimmer@uniklinik-freiburg.de (A.D.Z.); katalin.komlosi@uniklinik-freiburg.de (K.K.); nina.singh@uniklinik-freiburg.de (N.S.); andras.tzschach@uniklinik-freiburg.de (A.T.); judith.fischer@uniklinik-freiburg.de (J.F.); 2 Radiology, Medical Center, Faculty of Medicine, University of Freiburg, 79106 Freiburg, Germany; marisa.windfuhr-blum@uniklinik-freiburg.de; 3 Department of Obstetrics and Gynaecology, Medical Center, Faculty of Medicine, University of Freiburg, 79106 Freiburg, Germany; ingolf.juhasz-boess@uniklinik-freiburg.de (I.J.-B.); thalia.erbes@uniklinik-freiburg.de (T.E.)

**Keywords:** HBOC, *BRCA1*, *BRCA2*

## Abstract

In about 20–30% of all women with breast cancer, an increased number of cases of breast cancer can be observed in their family history. However, currently, only 5–10% of all breast cancer cases can be attributed to a pathogenic gene alteration. Molecular genetic diagnostics underwent enormous development within the last 10 years. Next-generation sequencing approaches allow increasingly extensive analyses resulting in the identification of additional candidate genes. In the present work, the germline molecular diagnostic analysis of a cohort of 228 patients with suspected hereditary breast and ovarian cancer syndrome (HBOC) was evaluated. The 27 pathogenic gene variants initially detected are listed, and their distribution in the high-risk *BRCA1* and *BRCA2* genes is presented in this study. In ten high-risk patients, in whom, to date, no pathogenic variant could be detected, an extended genetic analysis of previously not considered risk genes was performed. Three variants of uncertain significance and one pathogenic variant could be described. This proves the importance of extended analysis using current molecular genetic methods.

## 1. Introduction

Human genetic analyses are an integral part of everyday clinical practice due to their scope and importance for subsequent therapeutic decisions. The introduction of high-throughput analytical techniques allows increasingly comprehensive testing, which in turn contributes to a better medical understanding of the disease. Around 30% of all women with breast cancer in Germany have a family history of breast cancer. They thus fulfill the inclusion criteria for genetic testing regarding hereditary breast and ovarian cancer syndrome (HBOC) [1]. In hereditary breast and ovarian cancer syndrome, high-risk genes (*BRCA1*, *BRCA2*, *TP53*, and *PALB2*) are distinguished in addition to moderately penetrant risk genes (currently *ATM*, *BARD1*, *BRIP1*, *CDH1*, *CHEK2*, *RAD51C*, and *RAD51D*) [2]. In the scope of the German Consortium for Hereditary Breast and Ovarian Cancer, other candidate genes (e.g., *NBN*, *FANCM*, *XRCC2*, and *RECQL*) are currently co-analyzed for research purposes and their significance in contributing to breast and ovarian cancer is under investigation [3]. Women with a *BRCA1* or *BRCA2* pathogenic variant develop disease approximately 10 to 20 years earlier than women without familial risk [4]. Due to the increased lifetime risk of breast cancer of up to 70% [5], these women are eligible for an intensified screening and follow-up program [2].

We performed a comprehensive statistical evaluation in a cohort of 228 patients whose data were collected in 2012–2017 at the Institute of Human Genetics of the University Medical Center of Freiburg in the context of molecular genetic diagnostics regarding hereditary breast and ovarian cancer. The aim was to provide an overview of the overall spectrum and to identify relevant and previously undetected pathogenic variants in high-risk patients.

## 2. Materials and Methods

### 2.1. Data Collection and Risk Assessment

Initially, data were collected from 228 patients. These were fully considered in the statistical analysis. For 213 patients, detailed information of the family history was available, allowing an additional risk assessment using the checklist of the German Consortium for Hereditary Breast and Ovarian Cancer [6]. In addition to the age of onset of the disease, this checklist is based on other associated cases of the disease in the maternal and paternal line and takes these into account with a single, double, or triple weighting. For reasons of clarity and informative value, the score values obtained were grouped into specially determined subgroups (Table 1). This grouping is introduced and determined exclusively to differentiate the cohort under investigation.

### 2.2. Sequencing Methods and Bioinformatics Programs

The data collection of this work includes molecular genetic analyses from 2012 to 2017. The analysis of the high-risk genes *BRCA1* and *BRCA2* was performed using Sanger sequencing until August 2013. Subsequently, the analysis procedure was changed to next-generation sequencing (NGS). Then, in March 2015, there was another update from the BRCA Dx panel, which included only the *BRCA1* and *BRCA2* genes, to the BRCA Hc MASTR (both Multiplicom, Niel Belgium; now part of Agilent Technologies), which includes the genes *ATM*, *BARD1*, *BLM*, *BRCA1*, *BRCA2*, *BRIP1*, *CDH1*, *CHEK2*, *EPCAM*, *FAM175A*, *MEN1*, *MLH1*, *MRE11A*, *MSH2*, *MSH6*, *MUTYH*, *NBN*, *PALB2*, *PMS2*, *PTEN*, *RAD50*, *RAD51C*, *RAD51D*, *STK11*, *TP53*, and *XRCC2*. After joining the German Consortium for Hereditary Breast and Ovarian Cancer, the TruRisk® panel (Agilent Technologies) was launched in January 2019. It includes the 11 core genes *ATM*, *BARD1*, *BRCA1*, *BRCA2*, *BRIP1*, *CDH1*, *CHEK2*, *PALB2*, *RAD51C*, *RAD51D*, and *TP53*, as well as additional research genes, and is under continuous development. The extended molecular genetic analysis performed here using DNA isolated from peripheral blood, the TruRisk® panel, and an Illumina MiSeq system (2 × 150 base pairs, paired end; Illumina, San Diego, CA, US) yielded a mean target coverage for the eleven core genes of 405× with 99.9% of the bases covered more than 20×. In silico analyses to predict the pathogenicity of DNA variants have been performed using the following bioinformatics programs: Mutation Taster (http://www.mutationtaster.org/ accessed on 10 September 2021) [8], PolyPhen-2 (http://genetics.bwh.harvard.edu/pph2/ accessed on 12 May 2020) [9], FATHMM v2.3 (http://fathmm.biocompute.org.uk/ accessed on 12 May 2020) [10], SIFT (http://sift.jcvi.org/ accessed on 12 May 2020) [11], and NetGene2 v2.4 (http://www.cbs.dtu.dk/services/NetGene2/ accessed on 12 May 2020) [12]. Furthermore, the following databases were used: Genome Aggregation Database version v2.1.1 (gnomAD; http://gnomad.broadinstitute.org/ accessed on 12 May 2020), HGMD® Professional version 2020.3 (http://www.biobase-international.com/product/hgmd accessed on 12 May 2020), Database of Single Nucleotide Polymorphisms version build 151 (dbSNP; http://www.ncbi.nlm.nih.gov/projects/SNP/ accessed on 12 May 2020), PubMed (http://www.ncbi.nlm.nih.gov/pubmed/ accessed on 12 May 2020), and ClinVar (https://www.ncbi.nlm.nih.gov/clinvar/ accessed on 12 May 2020).

## 3. Results

### 3.1. Statistical Evaluation

The average age at analysis was 50.6 years for women and 57.4 years for men. At the time of analysis, 164 patients had breast cancer, 17 had ovarian cancer, and 7 had breast and ovarian cancer. A total of 27 people had predictive testing performed. For 13 patients, a reference to the respective background for the analysis performed was not possible, due to missing information. The classification of detected sequence variants was performed according to the standards and guidelines of the American College of Medical Genetics and Genomics (ACMG) [13].

In total, pathogenic variants (ACMG class 5) were detected in 37 (16.2%) patients. These were mainly found in the high-risk genes *BRCA1* (54.1%, *n* = 20) and *BRCA2* (37.8%, *n* = 14). In the *CHEK2* gene, two pathogenic variants were detected (5.4%, *n* = 2) and, in the *RAD51C* gene, one pathogenic variant was identified (2.7%, *n* = 1). In total, eleven patients with detected *BRCA1* variants had breast cancer, five patients had ovarian cancer, and two patients had breast and ovarian cancer. For two patients, no conclusion could be drawn about the cancer type due to missing information. Regarding *BRCA2* variants, twelve patients developed breast cancer. One patient had breast and ovarian cancer. For one patient, no conclusion could be drawn about the cancer type due to missing information. *BRCA1* mutation carriers are thus more likely to develop ovarian cancer than *BRCA2* mutation carriers, as previously reported [14,15]. All patients carrying a pathogenic *CHEK2* or *RAD51C* variant had breast cancer. All detected pathogenic variants are summarized with their respective reference in Table 2. The pathogenic variants c.5609_5610delTCinsA as well as c.7878G>C of the *BRCA2* gene were initially described in the context of Fanconi anemia disease. Later, they were associated with hereditary breast cancer. Each of the detected pathogenic variants had been described elsewhere before.

### 3.2. Graphical Representation of the Pathogenic Variants in the BRCA1 and BRCA2 Genes

**Figure 1:**
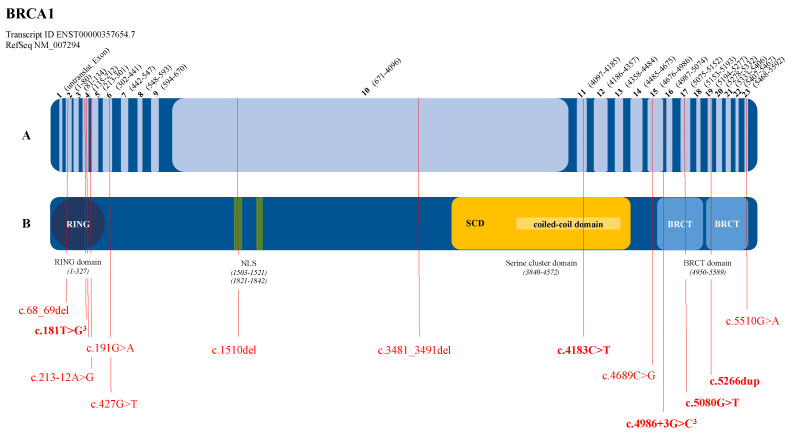
Localization of detected pathogenic variants in the *BRCA1* gene (RefSeq NM_007294). (**A**) Gene representation with exon and intron indication (light and dark blue, respectively)—bold numbers: exon numbering; numbers in parentheses: cDNA positions. (**B**) Protein representation—different colored shapes: functional domains; RING: really interesting new gene domain; NLS: nuclear localization sequence; SCD: serine cluster domain; BRCT: BRCA1 C-terminus domain. Pathogenic variants are indicated in red; bold notation of pathogenic variant: dual occurrence; superscript three: triple occurrence.

**Figure 2:**
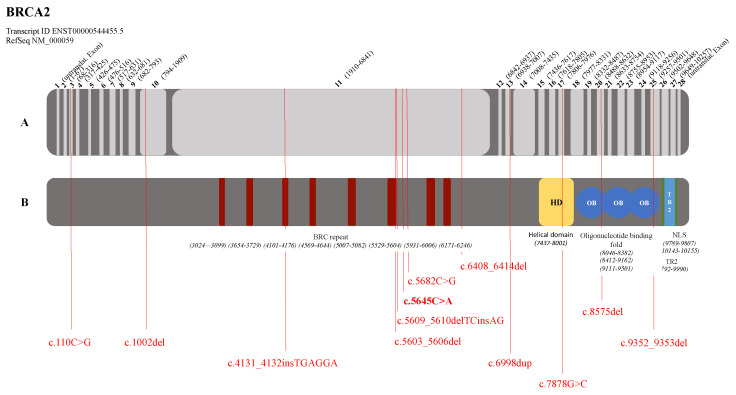
Localization of detected pathogenic variants in the *BRCA2* gene (RefSeq NM_000059). (**A**) Gene representation with exon and intron indication (light and dark grey, respectively)—bold numbers: exon numbering; numbers in parentheses: cDNA positions. (**B**) Protein representation—different colored shapes: functional domains; BRC repeat: BRC repeat domain; HD: helical domain; OB: oligonucleotide binding fold; NLS: nuclear localization sequence; TR2: tower domain. Pathogenic variants are indicated in red; bold notation of pathogenic variant: dual occurrence.

In the *BRCA1* gene, 70% of the pathogenic variants are located in the functional RING domain at the N-terminus (6 of the 20 pathogenic variants) or in the BRCT domains at the C-terminus (8 of the 20 pathogenic variants) (Figure 1). Furthermore, one pathogenic variant was detected in the nuclear localization sequence (c.1510del, p.(Arg504Valfs*28)) and another pathogenic variant (c.4183C>T, p.(Gln1395*)) in the coiled-coil domain in the region of the serine cluster (Figure 1). For the *BRCA2* gene, the pathogenic variants are mainly located in the BRC repeat domain (Figure 2), where 7 of the 14 detected pathogenic variants are found. Another four pathogenic variants are located in the functional domains at the C-terminus (Figure 2). Equal numbers of them are located in the helical domain and in the oligonucleotide binding fold (Figure 2).

Seventeen of the twenty detected pathogenic variants in the *BRCA1* gene (=85.0%) and eleven of the fourteen detected pathogenic variants in the *BRCA2* gene (=78.6%) can be localized in the conserved regions of the genes. These are essential for correct protein structure and function. Thus, in the presence of such a pathogenic variant in the case of a missense variant, a loss of function of the protein can be assumed.

### 3.3. Evaluation of the Molecular Genetic Analyses of the High-Risk Patient Group

Because of the further development of sequencing technologies, a renewed and extended gene analysis with the TruRisk® panel of the German Consortium of Hereditary Breast and Ovarian Cancer was performed for ten patients who showed a particularly high-familial-risk constellation without the detection of a pathogenic variant. As a result, four variants could be detected (Table 3).

Pathogenic *ATM* variants increase the risk for breast cancer by 2–3 times [42]. Evidence regarding an increased risk of prostate or pancreatic cancer cannot be confirmed currently due to insufficient data [43,44]. Pathogenic *BARD1* variants also lead to increased risk for Ewing sarcoma, osteosarcoma, and neuroblastoma, in addition to an increased risk for breast cancer [45,46,47]. The *MUTYH* and *SMARCA4* genes are among the candidate genes of the TruRisk® panel. In addition to *MUTYH*-associated polyposis (MAP) and the associated increased risk for colon cancer, pathogenic *MUTYH* variants have been described to confer increased risks for, among others, urinary bladder and gastric cancer [48]. Pathogenic variants in the *SMARCA4* gene are increasingly associated with small-cell carcinoma of the ovary, hypercalcemic type, or adenocarcinomas of the lung and endometrium [49,50,51].

The detected *ATM* variant is a class 5 pathogenic variant generating a premature stop codon. The databases HGMD® Professional 2020.3 and ClinVar contain several entries describing the variant as pathogenic [52,53]. For the *BARD1* variant c.212G>T, the sequence variant interpretation tools SIFT, Polyphen2, and MutationTaster predict a putative deleterious effect of the variant. In the ClinVar database, the variant was reported five times, most recently in November 2018, and interpreted with unclear significance in all cases. There is no entry in the HGMD® Professional 2020.3 database for this specific pathogenic variant, but there are two entries for other variants at the same position with a different nucleotide exchange (c.212G>A; c.212G>C). The variant c.212G>A p.(Cys71Tyr) RefSeq NM_000465.4 is described as a disease-causing variant, and the variant c.212G>C p.(Cys71Ser) RefSeq NM_000465.4 as a questionable pathogenic variant [54,55]. The patient in whom the pathogenic *ATM* variant and the *BARD1* variant of uncertain significance were detected had breast cancer at the age of 39 and ovarian cancer at the age of 48.

The *MUTYH* variant c.919C>T can also be classified as a class 3 variant. The programs SIFT, MutationTaster, and FATHMM indicate a probably deleterious effect of the variant, whereas Polyphen2 describes a possible deleterious effect.

The *SMARCA4* variant c.2275-3C>A is an intron variant with possible influence on the splicing process. It has been reported nine times in the ClinVar database with varying classifications, most recently in May 2019 as a probably benign variant. The prediction programs Human Splicing Finder version 3.1 and NNSplice indicated a most likely influence on the splicing process at the acceptor site. The program NetGene2 version 2.4 predicted no change in splicing efficiency. There is currently no entry for this variant in the HGMD database or in the German Consortium of Hereditary Breast and Ovarian Cancer BRCA2006 database.

## 4. Discussion

The further development of analytical methods opens up a multitude of new possibilities. The analysis of the present cohort shows that within a five-year period, the standard analysis method has evolved from Sanger sequencing to next-generation sequencing. This high rate of technical change results not only in a large amount of data with different sequence variants but also in a steady stream of new candidate genes, the clinical significance of which is sometimes still unclear at the time of analysis. A large data resource is needed to achieve an assessment of candidate genes in terms of their clinical relevance and to incorporate them into routine diagnostics. At the same time, a more comprehensive panel diagnosis increases the probability to detect a variant of clinical relevance. However, the more genes are sequenced, the more variants of uncertain significance can be identified, which in turn cannot be clearly classified clinically. Thus, the challenge lies in the risk calculation and the statement of clinical evaluation. The diagnostic range may change over a few years, and a possible extended second or third analysis, and a re-evaluation of already described varieties, based on current scientific knowledge, may be useful. A possible recommendation would be a routine review as well as a supplement of the molecular genetic diagnostics according to the current state of research, for example in a 5- to 10-year rhythm. Currently, mostly only case–control studies are available for interpretation, which could promote misinterpretation. In order for the statements to gain a higher level of evidence, further investigations are needed, for example, in the context of prospective cohort studies. These are another important aspect to illustrate the existing possibility of multifactorial inheritance and pathogenicity, which may be causative for the disease. In this context, a single variant would moderately increase the risk of disease, whereas the presence of additional variants could potentiate the risk of tumor development. In addition, with many pathogenic variants detected, there is not only an increased risk for breast or ovarian cancer, but also other associated tumor diseases are possible. The statement of a genotype–phenotype relationship is also becoming increasingly important. Thus, different ovarian cancer cluster regions (OCCRs) and breast cancer cluster region (BCCRs) can be formulated for both the *BRCA1* and *BRCA2* genes. These are characterized in this specific region by a significantly higher rate of breast or ovarian cancer compared to the other entity [56,57]. In addition to mutation position analysis, there are other considerations for predicting specific cancer risks. For example, the prospective cohort studies from the International BRCA1/2 Carrier Cohort Study (IBCCS), Breast Cancer Family Registry (BCFR), and Kathleen Cuningham Foundation Consortium for Research Into Familial Breast Cancer (kConFab) suggest that both family history and variant location in the gene influence cancer risk [58,59,60]. Further research is needed in this regard. The present cohort analysis shows that familial clustering of breast and ovarian cancer can be accounted for by analyzing additional risk genes. It is worthwhile to revisit the variants found after a certain period of time (e.g., 5–10 years) as well as to extend the analysis in unexplained cases to verify the results with the current state of science, even if only one patient out of 228 benefits from it.

## Figures and Tables

**Table 1:** Distribution of the risk assessment of 213 patients based on the checklist of the German Consortium for Hereditary Breast and Ovarian Cancer [7].

Risk Score	0	1	2	3	4	5	6	7	8	9	10	11	12	13	14	15	20
Number of patients	1	2	15	47	46	36	25	12	9	8	2	5	1	1	1	1	1
∑	18			154				41									
Percentage [%]	0.5	0.9	7.0	22.0	21.6	17.0	11.7	5.6	4.2	3.8	0.9	2.3	0.5	0.5	0.5	0.5	0.5
∑	8.4%			72.3%				19.3%									
Classification	Low Risk			Intermediate Risk				High Risk									

**Table 2:** Pathogenic variants in the *BRCA1*, *BRCA2*, *CHEK2*, and *RAD51C* genes [7].

Gene	DNA Level	Protein Level	Cancer Type	Reference
*BRCA1* (NM_007294.4)	c.68_69del	p.(Glu23Valfs*17)	BC, 1 patient	Struewing et al., 1995 [16]
	c.181T>G	p.(Cys61Gly)	BC, 3 patients	Friedman et al., 1994 [17]
	c.191G>A	p.(Cys64Tyr)	BC + OC, 1 patient	Couch et al., 1996 [18]
	c.213-12A>G		OC, 1 patient	Hoffman et al., 1998 [19]
	c.427G>T	p.(Glu143*)	BC, 1 patient	Shattuck-Eidens et al., 1997 [20]
	c.1510del	p.(Arg504Valfs*28)	BC + OC, 1 patient	Machackova et al., 2008 [21]
	c.3481_3491del	p.(Glu1161Phefs*3)	OC, 1 patient	Struewing et al., 1995 [16]
	c.4183C>T	p.(Gln1395*)	OC, 2 patients	Langston et al., 1996 [22]
	c.4689C>G	p.(Tyr1563*)	BC, 1 patient	Serova et al., 1996 [23]
	c.4986+3G>C	p.(Met1663Valfs*14)	BC, 2 patients	Adem et al., 2003 [24]
	c.5080G>T	p.(Glu1694*)	BC, 1 patient unknown, 1 patient	Shattuck-Eidens et al., 1997 [20]
	c.5266dup	p.(Gln1756Profs*74)	BC, 2 patients	Simard et al., 1994 [25]
	c.5510G>A	p.(Trp1837*)	OC, 1 patient	Couch et al., 1996 [18]
*BRCA2* (NM_000059.3)	c.110C>G	p.(Ser37*)	BC, 1 patient	Tung et al., 2015 [26]
	c.1002del	p.(His334Glnfs*15)	BC + OC, 1 patient	Borg et al., 2010 [27]
	c.4131_4132insTGAGGA	p.(Thr1378*)	BC, 1 patient	Delgado et al., 2002 [28]
	c.5603_5606del	p.(Asp1868Valfs*5)	BC, 1 patient	Li et al., 2019 [29]
	c.5609_5610delTCinsA	p.(Phe1870*)	BC, 1 patient	Howlett et al., 2002 (Fanconi Anemia) [30] Tea et al., 2014 (Breast Cancer) [31]
	c.5645C>A	p.(Ser1882*)	BC, 1 patient unknown, 1 patient	De Benedetti et al., 1998 [32]
	c.5682C>G	p.(Tyr1894*)	PC, 1 patient	Risch et al., 2001 [33]
	c.6408_6414del	p.(Asn2137Lysfs*29)	BC, 1 patient	Tamboom et al., 2010 [34]
	c.6998dup	p.(Pro2334Thrfs*6)	BC, 1 patient	Tedaldi et al., 2017 [35]
	c.7878G>C	p.(Trp2626Cys)	BC, 2 patients	Barber et al., 2005 (Fanconi Anemia) [36] Lindor et al., 2012 (Breast Cancer) [37]
	c.8575del	p.(Gln2859Lysfs*4)	BC, 1 patient	Martin et al., 2001 [38]
	c.9352_9353del	p.(Met3118Valfs*31)	BC, 1 patient	Sun et al., 2017 [39]
*CHEK2* (NM_007194.4)	c.1100del	p.(Thr367Metfs*15)	BC, 2 patients	Bell et al., 1999 [40]
*RAD51C*	Deletion Exon 5-9		BC, 1 patient	Schubert et al., 2019 [41]

BC = breast cancer; OC = ovarian cancer; PC = pancreatic cancer

**Table 3:** Identified DNA variants as a result of the extended genetic analysis [7].

Gene	Transcript ID/RefSeq	DNA Level	Protein Level	dbSNP	MAF	Classification ACMG
*ATM*	ENST00000675843.1/NM_000051.4	c.7327C>T	p.(Arg2443*)	rs121434220	3.983 × 10^−6^	Class 5
*BARD1*	ENST00000260947.9/NM_000465.4	c.212G>T	p.(Cys71Phe)	rs1064793959	3.185 × 10^−5^	Class 3
*MUTYH*	ENST00000456914.7/NM_001048174.2	c.919C>T	p.(Arg307Trp)	rs759822330	2.388 × 10^−5^	Class 3
*SMARCA4*	ENST00000344626.10/NM_003072.5	c.2275-3C>A		rs117611401	2.521 × 10^−3^	Class 3

## Data Availability

The data presented in this study are available in this paper, Resch et al., 2021.

## References

[1] Leitlinienprogramm Onkologie (Deutsche Krebsgesellschaft, Deutsche Krebshilfe, AWMF) S3-Leitlinie Früherkennung, Diagnose, Therapie Und Nachsorge Des Mammakarzinoms, Version 4.1, AWMF Registernummer: 032-045OL. http://www.leitlinienprogramm-onkologie.de/leitlinien/mammakarzinom/.

[2] Deutsches Konsortium Familiärer Brust-und Eierstockkrebs Standard Operating Procedures Im Deutschen Konsortium Familiärer Brust-Und Eierstockkrebs 2019. As of 28.05.2019; Annex to the consortium agreement.

[3] Die TruRisk® Genpanel-Analyse Bei Familiärem Brust-Und Eierstockkrebs. https://www.konsortium-familiaerer-brustkrebs.de/betreuungskonzept/molekulare-diagnostik/truriskr-genpanel-analyse/.

[4] Engel C., Zachariae S., Fischer C. (2015). Familiärer Brustkrebs—Empirische Erkrankungsrisiken und Risikoberechnungsmodelle. Med. Genet..

[5] Kuchenbaecker K.B., Hopper J.L., Barnes D.R., Phillips K.-A., Mooij T.M., Roos-Blom M.-J., Jervis S., Van Leeuwen F.E., Milne R.L., Andrieu N. (2017). Risks of breast, ovarian, and contralateral breast cancer for *BRCA1* and *BRCA2* mutation carriers. JAMA.

[6] Deutsches Konsortium Familiärer Brust- und Eierstockkrebs Checkliste Zur Risikoerfassung. https://www.konsortium-familiaerer-brustkrebs.de/informationen/checkliste-zur-risikoerfassung/.

[7] Resch L.D. (2020). Molekulargenetische Diagnostik Bei Erblichem Brust-und Eierstockkrebs.

[8] Schwarz J.M., Cooper D.N., Schuelke M., Seelow D. (2014). MutationTaster2: Mutation Prediction for the Deep-Sequencing Age. Nat. Methods.

[9] Adzhubei I.A., Schmidt S., Peshkin L., Ramensky V.E., Gerasimova A., Bork P., Kondrashov A.S., Sunyaev S.R. (2010). A method and server for predicting damaging missense mutations. Nat. Methods.

[10] Shihab H.A., Gough J., Mort M., Cooper D.N., Day I.N., Gaunt T.R. (2014). Ranking non-synonymous single nucleotide polymorphisms based on disease concepts. Hum. Genom..

[11] Ng P.C., Henikoff S. (2001). Predicting deleterious amino acid substitutions. Genome Res..

[12] Hebsgaard S.M., Korning P.G., Tolstrup N., Engelbrecht J., Rouzé P., Brunak S. (1996). Splice site prediction in Arabidopsis thaliana pre-mRNA by combining local and global sequence information. Nucleic Acids Res..

[13] Richards S., Aziz N., Bale S., Bick D., Das S., Gastier-Foster J., Grody W.W., Hegde M., Lyon E., Spector E. (2015). Standards and guidelines for the interpretation of sequence variants: A joint consensus recommendation of the American College of Medical Genetics and Genomics and the Association for Molecular Pathology. Genet. Med..

[14] Antoniou A.C., Pharoah P.D.P., McMullan G., Day N.E., Stratton M.R., Peto J., Ponder B.J., Easton D.F. (2002). A comprehensive model for familial breast cancer incorporating BRCA1, BRCA2 and other genes. Br. J. Cancer.

[15] Prat J., Ribé A., Gallardo A. (2005). Hereditary ovarian cancer. Hum. Pathol..

[16] Struewing J.P., Brody L.C., Erdos M.R., Kase R.G., Giambarresi T.R., Smith S.A., Collins F.S., Tucker M.A. (1995). Detection of eight BRCA1 mutations in 10 breast/ovarian cancer families, including 1 family with male breast cancer. Am. J. Hum. Genet..

[17] Friedman L.S., Ostermeyer E.A., Szabo C.I., Dowd P., Lynch E.D., Rowell S.E., King M.C. (1994). Confirmation of BRCA1 by analysis of germline mutations linked to breast and ovarian cancer in ten families. Nat. Genet..

[18] Couch F.J., Weber B.L. (1996). Mutations and polymorphisms in the familial early-onset breast cancer (BRCA1) gene. Breast cancer information core. Hum. Mutat..

[19] Hoffman J.D., Hallam S.E., Venne V.L., Lyon E., Ward K. (1998). Implications of a novel cryptic splice site in the BRCA1 gene. Am. J. Med. Genet..

[20] Shattuck-Eidens D., Oliphant A., McClure M., McBride C., Gupte J., Rubano T., Pruss D., Tavtigian S.V., Teng D.H., Adey N. (1997). BRCA1 sequence analysis in women at high risk for susceptibility mutations: Risk factor analysis and implications for genetic testing. JAMA.

[21] Machackova E., Foretova L., Lukesova M., Vasickova P., Navratilova M., Coene I., Pavlu H., Kosinova V., Kuklova J., Claes K. (2008). Spectrum and characterisation of BRCA1 and BRCA2 deleterious mutations in high-risk czech patients with breast and/or ovarian cancer. BMC Cancer.

[22] Langston A.A., Malone K.E., Thompson J.D., Daling J.R., Ostrander E.A. (1996). BRCA1 mutations in a population-based sample of young women with breast cancer. N. Engl. J. Med..

[23] Serova O., Montagna M., Torchard D., Narod S.A., Tonin P., Sylla B., Lynch H.T., Feunteun J., Lenoir G.M. (1996). A high incidence of BRCA1 mutations in 20 breast-ovarian cancer families. Am. J. Hum. Genet..

[24] Adem C., Reynolds C., Soderberg C.L., Slezak J.M., McDonnell S.K., Sebo T.J., Schaid D.J., Myers J.L., Sellers T.A., Hartmann L.C. (2003). Pathologic characteristics of breast parenchyma in patients with hereditary breast carcinoma, including BRCA1 and BRCA2 mutation carriers. Cancer.

[25] Simard J., Tonin P., Durocher F., Morgan K., Rommens J., Gingras S., Samson C., Leblanc J.F., Bélanger C., Dion F. (1994). Common origins of BRCA1 mutations in canadian breast and ovarian cancer families. Nat. Genet..

[26] Tung N., Battelli C., Allen B., Kaldate R., Bhatnagar S., Bowles K., Timms K., Garber J.E., Herold C., Ellisen L. (2015). Frequency of mutations in individuals with breast cancer referred for BRCA1 and BRCA2 testing using next-generation sequencing with a 25-gene panel. Cancer.

[27] Borg A., Haile R.W., Malone K.E., Capanu M., Diep A., Törngren T., Teraoka S., Begg C.B., Thomas D.C., Concannon P. (2010). Characterization of BRCA1 and BRCA2 deleterious mutations and variants of unknown clinical significance in unilateral and bilateral breast cancer: The WECARE study. Hum. Mutat..

[28] Delgado L., Fernández G., González A., Bressac-de Paillerets B., Gualco G., Bombled J., Cataldi S., Sabini G., Roca R., Musé I.M. (2002). Hereditary breast cancer associated with a germline BRCA2 mutation in identical female twins with similar disease expression. Cancer Genet. Cytogenet..

[29] Li J.-Y., Jing R., Wei H., Wang M., Xiaowei Q., Liu H., Jian L., Ou J.-H., Jiang W.-H., Tian F.-G. (2019). Germline mutations in 40 cancer susceptibility genes among Chinese patients with high hereditary risk breast cancer. Int. J. Cancer.

[30] Howlett N.G., Taniguchi T., Olson S., Cox B., Waisfisz Q., De Die-Smulders C., Persky N., Grompe M., Joenje H., Pals G. (2002). Biallelic inactivation of BRCA2 in fanconi anemia. Science.

[31] Tea M.-K.M., Kroiss R., Muhr D., Fuerhauser-Rappaport C., Oefner P., Wagner T.M., Singer C.F. (2014). Central European BRCA2 mutation carriers: Birth cohort status correlates with onset of breast cancer. Maturitas.

[32] De Benedetti V.M., Radice P., Pasini B., Stagi L., Pensotti V., Mondini P., Manoukian S., Conti A., Spatti G., Rilke F. (1998). Characterization of ten novel and 13 recurring BRCA1 and BRCA2 germline mutations in italian breast and/or ovarian carcinoma patients. Mutations in brief No. 178. Online. Hum. Mutat..

[33] Risch H.A., McLaughlin J.R., Cole D.E., Rosen B., Bradley L., Kwan E., Jack E., Vesprini D.J., Kuperstein G., Abrahamson J.L. (2001). Prevalence and penetrance of germline BRCA1 and BRCA2 mutations in a population series of 649 women with ovarian cancer. Am. J. Hum. Genet..

[34] Tamboom K., Kaasik K., Aršavskaja J., Tekkel M., Lilleorg A., Padrik P., Metspalu A., Veidebaum T. (2010). BRCA1 mutations in women with familial or early-onset breast cancer and BRCA2 mutations in familial cancer in Estonia. Hered. Cancer Clin. Pract..

[35] Tedaldi G., Tebaldi M., Zampiga V., Danesi R., Arcangeli V., Ravegnani M., Cangini I., Pirini F., Petracci E., Rocca A. (2017). Multiple-gene panel analysis in a case series of 255 women with hereditary breast and ovarian cancer. Oncotarget.

[36] Barber L.M., Barlow R.A., Meyer S., White D.J., Will A.M., Eden T.O.B., Taylor G.M. (2005). Inherited FANCD1/BRCA2 exon 7 splice mutations associated with acute myeloid leukaemia in Fanconi anaemia D1 are not found in sporadic childhood leukaemia. Br. J. Haematol..

[37] Lindor N.M., Guidugli L., Wang X., Vallée M.P., Monteiro A.N.A., Tavtigian S., Goldgar D.E., Couch F.J. (2012). A review of a multifactorial probability-based model for classification of BRCA1 and BRCA2 variants of uncertain significance (VUS). Hum. Mutat..

[38] Martin A.M., Blackwood M.A., Antin-Ozerkis D., Shih H.A., Calzone K., Colligon T.A., Seal S., Collins N., Stratton M.R., Weber B.L. (2001). Germline mutations in BRCA1 and BRCA2 in breast-ovarian families from a breast cancer risk evaluation clinic. J. Clin. Oncol..

[39] Sun J., Meng H., Yao L., Lv M., Bai J., Zhang J., Wang L., Ouyang T., Li J., Wang T. (2017). Germline mutations in cancer susceptibility genes in a large series of unselected breast cancer patients. Clin. Cancer Res..

[40] Bell D.W., Varley J.M., Szydlo T.E., Kang D.H., Wahrer D.C., Shannon K.E., Lubratovich M., Verselis S.J., Isselbacher K.J., Fraumeni J.F. (1999). Heterozygous germ line HCHK2 mutations in Li-Fraumeni syndrome. Science.

[41] Schubert S., Van Luttikhuizen J.L., Auber B., Schmidt G., Hofmann W., Penkert J., Davenport C.F., Hille-Betz U., Wendeburg L., Bublitz J. (2019). The identification of pathogenic variants in BRCA1/2 negative, high risk, hereditary breast and/or ovarian cancer patients: High frequency of FANCM pathogenic variants. Int. J. Cancer.

[42] Marabelli M., Cheng S.-C., Parmigiani G. (2016). Penetrance of ATM gene mutations in breast cancer: A meta-analysis of different measures of risk. Genet. Epidemiol..

[43] Schumacher F.R., Al Olama A.A., Berndt S.I., Benlloch S., Ahmed M., Saunders E.J., Dadaev T., Leongamornlert D., Anokian E., Cieza-Borrella C. (2018). Association analyses of more than 140,000 men identify 63 new prostate cancer susceptibility loci. Nat. Genet..

[44] Roberts N.J., Jiao Y., Yu J., Kopelovich L., Petersen G.M., Bondy M.L., Gallinger S., Schwartz A.G., Syngal S., Cote M.L. (2012). ATM mutations in patients with hereditary pancreatic cancer. Cancer Discov..

[45] Brohl A.S., Patidar R., Turner C.E., Wen X., Song Y.K., Wei J.S., Calzone K.A., Khan J. (2017). Frequent inactivating germline mutations in DNA repair genes in patients with Ewing sarcoma. Genet. Med..

[46] Ballinger M.L., Goode D.L., Ray-Coquard I., James P.A., Mitchell G., Niedermayr E., Puri A., Schiffman J.D., Dite G.S., Cipponi A. (2016). Monogenic and polygenic determinants of sarcoma risk: An international genetic study. Lancet Oncol..

[47] Pugh T.J., Morozova O., Attiyeh E.F., Asgharzadeh S., Wei J.S., Auclair D., Carter S.L., Cibulskis K., Hanna M., Kiezun A. (2013). The Genetic landscape of high-risk neuroblastoma. Nat. Genet..

[48] Win A.K., Reece J.C., Dowty J.G., Buchanan D.D., Clendenning M., Rosty C., Southey M.C., Young J.P., Cleary S.P., Kim H. (2016). Risk of extracolonic cancers for people with biallelic and monoallelic mutations in MUTYH. Int. J. Cancer.

[49] Tischkowitz M., Huang S., Banerjee S., Hague J., Hendricks W.P.D., Huntsman D.G., Lang J.D., Orlando K.A., Oza A.M., Pautier P. (2020). Small-Cell Carcinoma of the Ovary, Hypercalcemic Type-Genetics, New Treatment Targets, and Current Management Guidelines. Clin. Cancer Res..

[50] Schoenfeld A.J., Bandlamudi C., Lavery J.A., Montecalvo J., Namakydoust A., Rizvi H., Egger J., Concepcion C.P., Paul S., Arcila M.E. (2020). The genomic landscape of SMARCA4 alterations and associations with outcomes in patients with lung Cancer. Clin. Cancer Res..

[51] Kolin D.L., Quick C.M., Dong F., Fletcher C.D.M., Stewart C.J.R., Soma A., Hornick J.L., Nucci M.R., Howitt B.E. (2020). SMARCA4-deficient uterine sarcoma and undifferentiated endometrial carcinoma are distinct clinicopathologic entities. Am. J. Surg. Pathol..

[52] Lhota F., Zemankova P., Kleiblova P., Soukupova J., Vocka M., Stranecky V., Janatova M., Hartmannova H., Hodanova K., Kmoch S. (2016). Hereditary truncating mutations of DNA repair and other genes in BRCA1/BRCA2/PALB2-negatively tested breast cancer patients. Clin. Genet..

[53] Carter N.J., Marshall M.L., Susswein L.R., Zorn K.K., Hiraki S., Arvai K.J., Torene R.I., McGill A.K., Yackowski L., Murphy P.D. (2018). Germline pathogenic variants identified in women with ovarian tumors. Gynecol. Oncol..

[54] Lee C., Banerjee T., Gillespie J., Ceravolo A., Parvinsmith M.R., Starita L., Fields S., Toland A.E., Parvin J.D. (2015). Functional analysis of BARD1 missense variants in homology directed repair of DNA double strand breaks. Hum. Mutat..

[55] Weber-Lassalle N., Borde J., Weber-Lassalle K., Horváth J., Niederacher D., Arnold N., Kaulfuß S., Ernst C., Paul V.G., Honisch E. (2019). Germline loss-of-function variants in the BARD1 gene are associated with early-onset familial breast cancer but not ovarian cancer. Breast Cancer Res..

[56] Thompson D., Easton D., Breast Cancer Linkage Consortium (2002). Variation in BRCA1 Cancer Risks by Mutation Position. Cancer Epidemiol. Biomark. Prev..

[57] Rebbeck T.R., Mitra N., Wan F., Sinilnikova O.M., Healey S., McGuffog L., Mazoyer S., Chenevix-Trench G., Easton D.F., Antoniou A.C. (2015). Association of type and location of BRCA1 and BRCA2 mutations with risk of breast and ovarian cancer. JAMA.

[58] Antoniou A.C., Goldgar D.E., Andrieu N., Chang-Claude J., Brohet R., Rookus M.A., Easton D.F. (2005). A weighted cohort approach for analysing factors modifying disease risks in carriers of high-risk susceptibility genes. Genet. Epidemiol..

[59] Terry M.B., Liao Y., Whittemore A.S., Leoce N., Buchsbaum R., Zeinomar N., Dite G.S., Chung W.K., Knight J.A., Southey M.C. (2019). 10-year performance of four models of breast cancer risk: A validation study. Lancet Oncol..

[60] Kramer I., Hooning M.J., Mavaddat N., Hauptmann M., Keeman R., Steyerberg E.W., Giardiello D., Antoniou A.C., Pharoah P.D.P., Canisius S. (2020). Breast cancer polygenic risk score and contralateral breast cancer risk. Am. J. Hum. Genet..

